# Adaptation and Implementation of a Mobile Phone–Based Remote Symptom Monitoring System for People With Cancer in Europe

**DOI:** 10.2196/10813

**Published:** 2019-03-14

**Authors:** Eileen Furlong, Andrew Darley, Patricia Fox, Alison Buick, Grigorios Kotronoulas, Morven Miller, Adrian Flowerday, Christine Miaskowski, Elisabeth Patiraki, Stylianos Katsaragakis, Emma Ream, Jo Armes, Alexander Gaiger, Geir Berg, Paul McCrone, Peter Donnan, Lisa McCann, Roma Maguire

**Affiliations:** 1 School of Nursing, Midwifery and Health Systems University College Dublin Dublin Ireland; 2 School of Psychological Sciences and Health University of Strathclyde Glasgow United Kingdom; 3 Docobo Ltd Surrey United Kingdom; 4 Institute for Global Health Sciences University of California San Francisco San Francisco, CA United States; 5 National and Kapodistrian University of Athens Athens Greece; 6 School of Health Sciences University of Surrey Surrey United Kingdom; 7 Division of Hematology and Hemaostaseology Medical University of Vienna Vienna Austria; 8 Faculty of Medicine and Health Sciences Norwegian University of Science and Technology Gjøvik Norway; 9 Innlandet Hospital Trust Division Lillehammer Lillehammer Norway; 10 Institute of Psychiatry, Psychology & Neuroscience King’s College London London United Kingdom; 11 Dundee Epidemiology and Biostatistics Unit University of Dundee Dundee United Kingdom

**Keywords:** telemedicine, methods, patient care, cancer, symptom management

## Abstract

**Background:**

There has been an international shift in health care, which has seen an increasing focus and development of technological and personalized at-home interventions that aim to improve health outcomes and patient-clinician communication. However, there is a notable lack of empirical evidence describing the preparatory steps of adapting and implementing technology of this kind across multiple countries and clinical settings.

**Objective:**

This study aimed to describe the steps undertaken in the preparation of a multinational, multicenter randomized controlled trial (RCT) to test a mobile phone–based remote symptom monitoring system, that is, Advanced Symptom Management System (ASyMS), designed to enhance management of chemotherapy toxicities among people with cancer receiving adjuvant chemotherapy versus standard cancer center care.

**Methods:**

There were 13 cancer centers across 5 European countries (Austria, Greece, Ireland, Norway, and the United Kingdom). Multiple steps were undertaken, including a scoping review of empirical literature and clinical guidelines, translation and linguistic validation of study materials, development of standardized international care procedures, and the integration and evaluation of the technology within each cancer center.

**Results:**

The ASyMS was successfully implemented and deployed in clinical practices across 5 European countries. The rigorous and simultaneous steps undertaken by the research team highlighted the strengths of the system in clinical practice, as well as the clinical and technical changes required to meet the diverse needs of its intended users within each country, before the commencement of the RCT.

**Conclusions:**

Adapting and implementing this multinational, multicenter system required close attention to diverse considerations and unique challenges primarily related to communication and clinical and technical issues. Success was dependent on collaborative and transparent communication among academics, the technology industry, translation partners, patients, and clinicians as well as a simultaneous and rigorous methodological approach within the 5 relevant countries.

## Introduction

### Background

The expanding field of electronic health (eHealth) and the global deployment of technology within health care have become more apparent over 20 years of research [1-4]. The increase in technological capabilities has led to many promising eHealth advancements in the cancer setting. For instance, an increasing number of health care initiatives in cancer care have utilized patients’ self-reports to facilitate remote symptom monitoring [5-11]. With regard to conducting empirical research on this scale in this field, there is an increasing awareness of the importance of preliminary work in preparation for large publicly funded randomized controlled trials (RCTs) [12]. This preliminary study allows a research team to make judgments about an eHealth system, and such preparation can facilitate researcher readiness for full-scale implementation [13]. While multinational research to evaluate the effectiveness of eHealth may present several opportunities and important findings, there are also a number of challenges and considerations when conducting research involving multiple countries, including differences in clinical settings such as resources and workflow, language and translation issues, as well as cultural and societal differences [14].

Moreover, conducting cross-cultural, multinational eHealth research requires collaboration and multiple considerations to ensure an eHealth system’s validity, fidelity, and appropriateness within different cultural and clinical settings [15-17]. This paper seeks to address an important gap in knowledge regarding the steps involved in adapting an eHealth system within cancer care across multiple countries. This gap may be in part because of the fact that eHealth remains a relatively new area of research characterized by exploratory studies implementing novel technology in cancer care practice and assessing their feasibility in a single country [18-22].

In this paper, the steps employed to adapt and implement a mobile phone–based remote symptom monitoring system, the Advanced Symptom Management System (ASyMS), into European cancer care before its deployment in a multinational RCT involving 13 cancer centers across 5 countries (ie, Norway, Austria, Greece, Ireland, and the United Kingdom) are described. We detail the robust, structured, and systematic approach to the adaption of the system and its controlled implementation at multiple cancer centers across Europe. The valuable points of learning arising from implementing this unique eHealth system on such a large multinational scale for future researchers will also be discussed.

### Advanced Symptom Management System

The ASyMS is an eHealth system that has undergone several years of testing, development, and evaluation [23-29] to monitor and help patients with cancer manage their chemotherapy-related symptoms at home. Although previously tested and studied in the United Kingdom, the ASyMS is currently being studied at a multinational level for the first time as an RCT—study title: *Electronic Symptom Management using Advanced Symptom Management System (ASyMS) Remote Technology for Patients With Cancer (eSMART)*. The protocol for the study has been previously published [30]. The purpose of undertaking the steps described in this paper was to examine and ensure cancer centers’ technological readiness before commencing the RCT.

**Figure 1:**
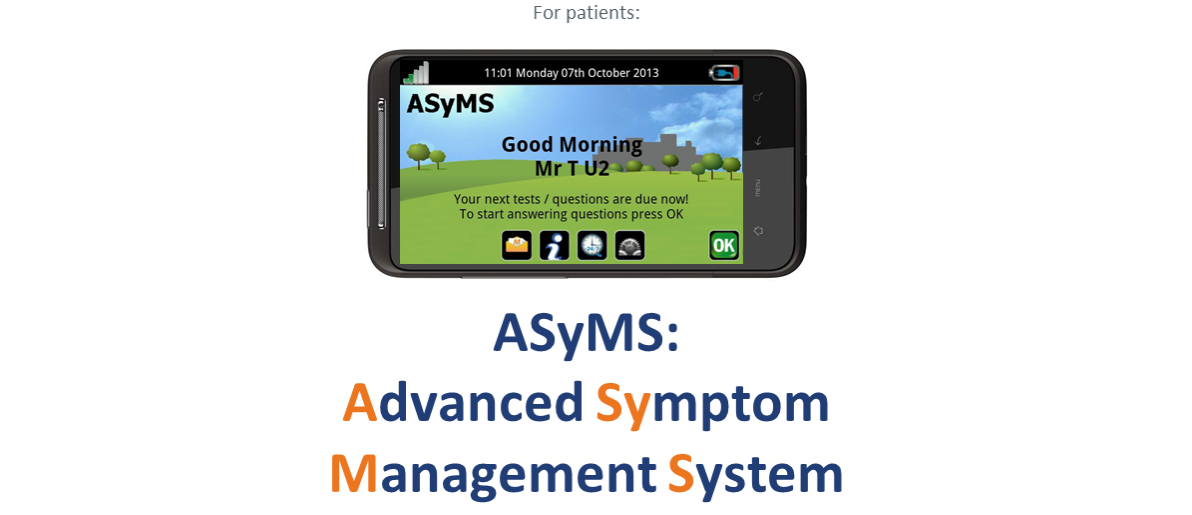
The Advanced Symptom Management System patient handset.

The ASyMS is a purpose-built, mobile phone–based remote symptom monitoring system to enable real-time, 24-hour monitoring and management of patients’ self-reported chemotherapy-related toxicities. The ASyMS is hosted by the eSMART Consortium technological partner, Docobo. The core component of the ASyMS is the mobile phone device, that is, the ASyMS patient handset (Figure 1).

**Figure 2:**
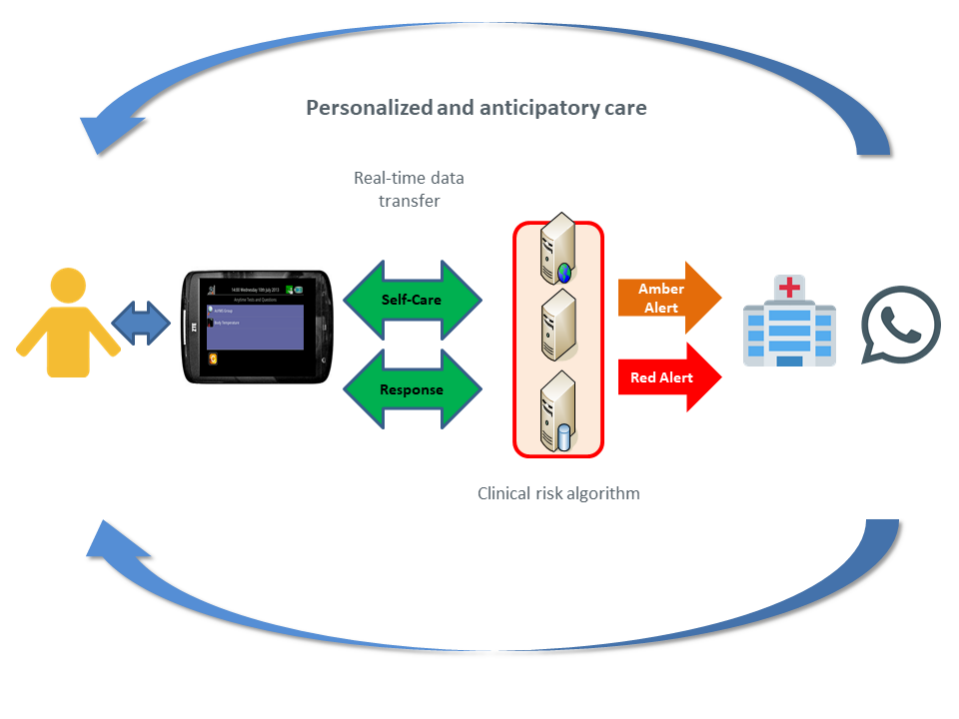
The Advanced Symptom Management System care pathway.

Patients are required to complete a symptom questionnaire—Chemotherapy Toxicity Self-Assessment Questionnaire—once a day, which is a patient-related outcome measure, developed by the ASyMS research team to facilitate rapid and accurate daily assessments of chemotherapy toxicity in clinical practice [31]. The questionnaire assesses 10 specific chemotherapy-related symptoms (ie, nausea, vomiting, diarrhea, constipation, hand-foot syndrome, mucositis, paresthesia, flu-like symptoms, fatigue, and pain). Additionally, if patients’ existing symptoms escalate or new symptoms are experienced, they can be reported using the ASyMS patient handset. The ASyMS analyses the information using an integrated clinical risk algorithm, as shown in The ASyMS Care Pathway (Figure 2), which initiates an alert to the clinical team at the patient’s cancer center. The ASyMS involves 3 types of alerts [30]:

1. A *green alert* is activated when a patient reports symptom that can be managed at home, without requiring current clinical intervention, using self-care advice by a clinician.
2. An *amber alert* is sent to a clinician if patients’ symptoms are bordering on becoming problematic and are responsive to early preventative interventions. Amber alerts are to be addressed within 8 hours by a clinician.
3. A *red alert* is sent to a clinician if patients’ symptoms are severe or life-threatening. Red alerts are to be addressed within 30 minutes.

**Figure 3:**
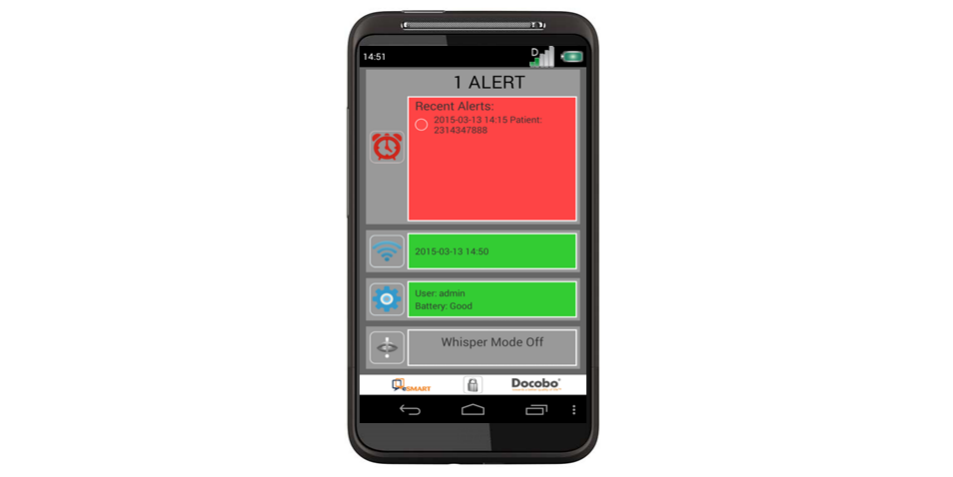
The Advanced Symptom Management System clinician handset.

The ASyMS patient handset contains an in-built library that generates self-care advice each time a patient completes the questionnaire, specific to the experienced symptoms. The self-care library and graphical depiction of their symptoms can be viewed by patients at any time.

For any symptom that requires clinical intervention (amber or red alerts), the algorithm generates *real-time* alerts to the cancer center via a dedicated ASyMS clinician handset (Figure 3). This specialized mobile phone–based clinician handset is carried by an alert handler (ie, clinician) at all times to receive alerts. Once an alert is received, the alert handler views the patient’s *real-time* symptom reports on a secure stand-alone ASyMS clinician website before contacting the patient to initiate the appropriate care intervention.

Alert handlers can access patients’ symptom reports, demographic and clinical information, contact telephone numbers, and addresses to facilitate an initial telephone assessment with the patient. Clinicians can store summaries of alert outcomes in the patients’ local medical records. Clinical algorithms based on international, national, and local guidelines as well as feedback from clinicians and patients determine the appropriate standardized interventions for the type of alert generated. The alert handler documents the intervention in the patients’ clinical case notes.

## Methods

In preparation for the use of the ASyMS within a multinational, multicenter RCT, the following steps were undertaken.

### Scoping Review

Although the ASyMS was rigorously developed and empirically studied previously in the United Kingdom [23-29], in order to upscale the system to various European countries, a scoping review was undertaken to ensure that it is consistent with international, national, and relevant local guidelines for assessing and managing the most common chemotherapy-related symptoms. This review that included evidence on the management of chemotherapy toxicity within Europe (assessment, management, and self‐care) was published [32]. Following the scoping review, the assessment, management interventions (including responses to alerts), and self‐care for the ASyMS were agreed upon by the research team using a consultation exercise undertaken with clinicians (clinical advisory group) and patients (patient advisory group) at the participating cancer centers to ensure standardized practice across all cancer centers.

### Translation and Linguistic Validation of the Advanced Symptom Management System Materials

Given that the ASyMS would be used simultaneously within 5 different European countries, it was paramount that all the study materials were translated and validated linguistically for use in non-English speaking countries. The ASyMS and all related study documents were to be available in German, Norwegian, and Greek. Although a majority of outcome measures were previously available in the language of the participating countries, some required translation for their use in the ASyMS. The International Society for Pharmacoeconomics and Outcomes Research (ISPOR) Patient-Reported Outcomes Translation and Linguistic Validation Task Force guidelines [33] were used to guide the translation and validation process. Included in the translation process were the following:

- The ASyMS clinician website
- The ASyMS patient handset
- The ASyMS clinician handset
- The ASyMS technical support website
- Patient-reported outcome measures
- Additional data collection forms and questionnaires
- Supporting documentation, including the study protocol, patient and clinician documents, and user manuals.
- eSMART research project website

The 2 key components of the translation process were (1) translation and linguistic validation of questionnaires into the required languages for the participating cancer centers and (2) translation of all additional study components and supporting documentation into the required languages (eg, patient information letters and consent forms). The employment of a translation company was necessary to complete this step. A total of 4 translation companies were evaluated to undertake this task based on the following criteria:

1. Compliance with ISPOR translation and validation guidelines
2. Experience in the translation and validation of patient-reported outcome measures as documented through previous collaborations and completed projects beforehand
3. Documented reliability and trustworthiness based on testimonials
4. Acceptable costs and turnaround times to ensure project cost-effectiveness

Following this evaluation process, Language Scientific was the chosen company that translated and linguistically validated the ASyMS questionnaires based on the robustness of their approach and costs.

### Preparation and Evaluation of Cancer Centers for the Use of the Advanced Symptom Management System

The preparation and evaluation of the cancer centers for the use of the ASyMS required an assessment of their technology infrastructure and human and material resource requirements. As the ASyMS required simultaneous implementation within 13 cancer centers in 5 countries, monthly teleconferences were held with all study partners to provide an opportunity to inform, assess progress, update, and identify any issues in this step. The teleconferences were attended by representatives in all partner countries, which facilitated open discussions and necessary actions around issues including ethics and governance, data protection, study instruments, technology development, and language translation processes. Additionally, clinicians and researchers committed to and participated in monthly teleconference meetings which were well-attended at this stage of implementation to discuss practical, clinical, and technical issues of using the ASyMS at each cancer center.

Before the selection of each cancer center to participate in the RCT, the reliability of Wi-Fi and mobile data networks was assessed at each cancer center. This assessment was conducted by Docobo using a Connectivity Logger app, which was run on Motorola Moto g mobile handsets at each of the participating cancer centers. All the handsets were procured by Docobo, marked with an individual tracking number, uploaded with the ASyMS, and distributed to each cancer center. Each research nurse, clinician, and research assistant was provided with training on the ASyMS, this included education regarding how the ASyMS works, patient registration, and alert handling. They were then registered with individual log-ins on the ASyMS, with the appropriate functions of patient registration and alert handling. Subsequently, researchers at each cancer center managed the handsets and provided them to the patients when recruited to the feasibility study.

An assessment of the ASyMS technological readiness with cancer care practice was necessary before its use in the RCT. This was undertaken with a small sample (n=64) of the intended population for the RCT across the 13 cancer centers. Data captured (eg, patient completion of the daily questionnaire, clinician initial response times to alerts, and clinician handling times of alerts) were used to assess the readiness of each cancer center to begin the RCT. All feasibility data were extracted from the study’s secure database hosted by Docobo. Technological readiness was assessed and confirmed using 2 Technological Feasibility Evaluation forms developed by the study investigators—1 for clinicians using the ASyMS (Multimedia Appendix 1) and 1 for Docobo (Multimedia Appendix 2) to complete. The 3 key parameters of technological readiness set out in the study protocol were examined:

1. *System set-up*: to assess whether clinicians and researchers had received sufficient training on the ASyMS, were able to register participants to use the ASyMS (using handset, tablet, and personal computer), and were confident to educate and register a new patient on a handset.
2. *Data transfer*: to assess whether data were successfully transferred between the ASyMS patient and clinician handsets, electronic clinical case note reviews, and the study server. It was essential that all handsets (ie, patient handsets and clinician handsets) had the required mobile or Wi-Fi connectivity for the intervention to be safe and effective.
3. *Usability issues*: to assess whether the patients could use the ASyMS patient handset, as well as the clinicians’ ability to use the ASyMS clinician handset, log on to the ASyMS clinician website, handle patient alerts, and complete medical reviews at the end of the patients’ chemotherapy cycle. The ASyMS technical support website, from both the clinician’s and patient’s perspective, was also evaluated.

On completion of the technological readiness assessment at each cancer center, a representative from the cancer center and the technological partner were required to complete their respective Technological Feasibility Evaluation forms, which were subsequently checked by the ASyMS research team for any discrepancies that needed to be addressed.

## Results

### The Findings

ASyMS was successfully adapted and implemented at 11 cancer centers across 5 European countries. The system was fully prepared for its deployment at each cancer center in providing care to their patients before commencing its large-scale RCT. The findings from each step of the adaption and implementation process will now be outlined.

### Scoping Review

The findings from the scoping review were used to update the self-care advice within the ASyMS and refine the clinical risk algorithms for the alerting system. Following the completion of the scoping review, a consultation exercise was undertaken with clinicians (clinical advisory group) and patients (patient advisory group) at the participating cancer centers [32]. The review found discrepancies among the published literature and the clinical advisory groups regarding the treatment of febrile neutropenia (fever) and what temperature rating was considered to warrant medical attention. It was concluded to use the most conservative scenario for safety reasons (37.5 Celsius) [32].

### Translation and Validation of Study Materials

The questionnaires and related documents involved in the ASyMS were successfully translated into the required languages. Minor queries were raised by the chosen translation company regarding specific items on the study questionnaires for Greek and Norwegian translation. The company sought confirmation from the research team to proceed with slight modifications of questionnaire items based on the feedback from the cognitive debriefing participants to ensure that they were culturally appropriate.

The translation involved 3 translation rounds and interviews with lay people in the respective countries (Austria, Greece, and Norway) in accordance with the current guidelines outlined by the ISPOR [33], which involved forward and back translation. For each component of the ASyMS, the information technology interface and documentation were adapted and translated for clinical use. Once the intervention content was translated and validated, ethical approval was obtained from the relevant ethics committees in all of the cancer centers across the 5 participating countries, as detailed in the protocol publication [30].

### Assessment of the Cancer Centers’ Technological Infrastructure

A crucial component of the implementation of the ASyMS at cancer centers was the assessment of technological readiness, which was undertaken by Docobo. The Connectivity Logger app, installed on the ASyMS clinician handset, measured and logged the quality of mobile and Wi-Fi networks at 1-min intervals while the handset was being carried by clinicians during their working hours. Areas in a cancer center where the clinician handset could not access Wi-Fi or a mobile data network were identified. The connectivity information was analyzed by Docobo. Clinicians were required to log at least 12 hours of mobile data and Wi-Fi.

The primary criterion for the connectivity assessment was the maximum sustained period for which no communication over the mobile network (ie, neither mobile internet protocol or text communications) was possible, being no more than 15 minutes (target response time was 30 minutes). The secondary factors considered were the distribution of signal strength and the quality of the mobile data connection. Analysis showed that at most cancer centers, the connectivity environment was favorable in providing a reliable communication channel to the ASyMS clinician handset. However, 1 cancer center had a loss of connectivity for up to 20 minutes (based on 800 hours of testing) compared with other cancer centers that had between 5 and 12 min of lack of connectivity. The Docobo team visited the cancer center to investigate the cause and concluded that the lack of connectivity occurred in the corridors of the cancer center and not on the relevant oncology ward, where suboptimal connectivity forced the handset to connect to a weak mobile network. Given the potential impact on clinical care should an alert not be received on time because of lack of connectivity, all clinician handsets needed to monitor for and make clinicians aware of a loss of network connectivity. Changes were made to the ASyMS, which could monitor the clinician handset at all times and make clinicians aware, via automated short message service text messaging and email, when a handset lost connectivity. It was concluded that 2 active handsets were necessary at each cancer center, with one in use and the second on charge, to allow for efficient charging and thus ensuring clinicians could hold the handset with 24-hour coverage as required.

### Feasibility Study of the Advanced Symptom Management System at European Cancer Centers

A total of 13 cancer centers across 5 European countries (ie, Austria, Greece, Ireland, Norway, and the United Kingdom) participated in the feasibility study. During this testing phase, 64 patients consented to use the ASyMS over 1 cycle of chemotherapy. At each cancer center, 2 patients per cancer type (not all cancer centers included all 3 patient populations) were recruited to test the system. Inclusion and exclusion criteria are detailed in Textbox 1 and Textbox 2, and patient numbers per diagnosis at the different European cancer centers are shown in Table 1.

**Participant eligibility inclusion criteria.**

- Adults (≥18 years)
- Diagnosed with breast cancer, colorectal cancer, Hodgkin’s disease, or non-Hodgkin’s lymphoma
- Currently receiving or about to start first-line chemotherapy
- Scheduled to receive 2, 3, or 4 weekly chemotherapy protocols (ie, chemotherapy administered every 14, 21, or 28 days, respectively)
- Scheduled to receive 1 cycle of chemotherapy
- Physically or psychologically fit to participate in the study
- Able to understand and communicate in the respective language

**Participant eligibility exclusion criteria.**

- Diagnosed with a distant metastasis in the case of breast cancer or colorectal cancer
- Experiencing B symptoms in the context of a Hodgkin’s disease or non-Hodgkin’s lymphoma diagnosis
- Scheduled to receive concurrent radiotherapy
- Scheduled to receive weekly chemotherapy
- Diagnosed with recurrent cancer
- Patients who have had chemotherapy within the previous 5 years for any medical reason
- Unable to provide written informed consent

**Table 1:** Patients recruited to conduct the feasibility study at each cancer center.

Study Center	Breast, n	Colorectal, n	Hematological, n
Cancer Center 1: Austria	2	2	2
Cancer Center 2: United Kingdom	2	2	1
Cancer Center 3: United Kingdom	2	2	—^a^
Cancer Center 4: United Kingdom	2	2	2
Cancer Center 5: United Kingdom	2	2	2
Cancer Center 6: Greece	2	2	—
Cancer Center 7: Greece	2	2	—
Cancer Center 8: Greece	2	2	2
Cancer Center 9: Ireland	2	2	—
Cancer Center 10: Ireland	2	2	—
Cancer Center 11: Ireland	2	2	2
Cancer Center 12: Ireland	2	2	1
Cancer Center 13: Norway	2	2	—
Total	26	26	12

^a^These sites did not recruit participants with hematological cancer.

#### Participants

**Figure 4:**
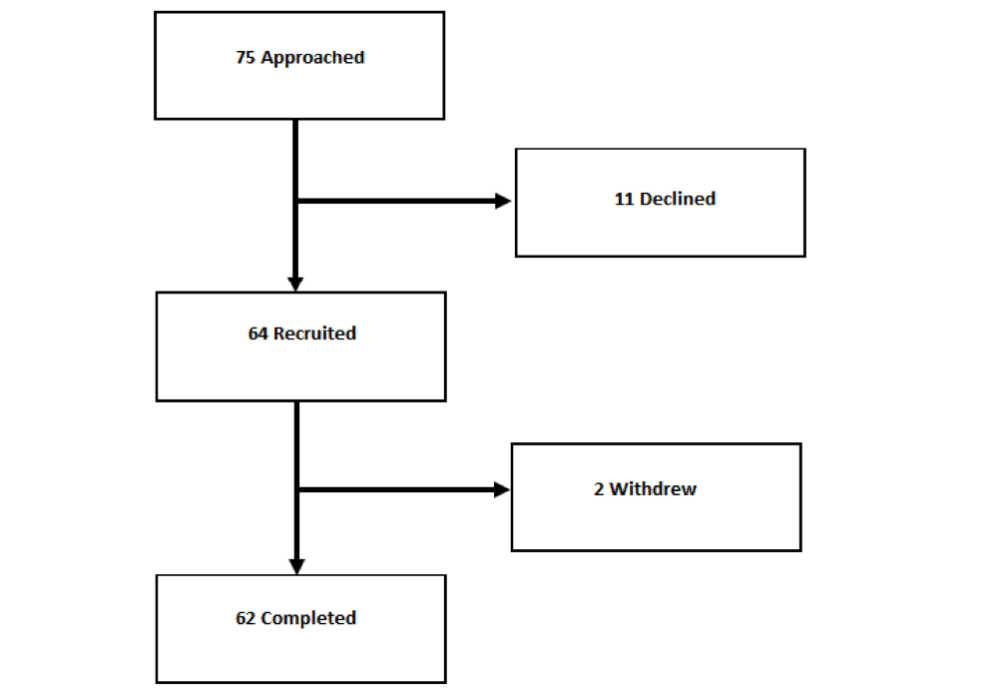
Patient recruitment.

Data on the testing of the ASyMS at each cancer center were collated by Docobo and analyzed by the members of the author team. Across all cancer centers, 85% (64/75) of eligible patients agreed to participate (Figure 4). Those who declined to participate cited being too busy, feared the study would increase worry and stress about the diagnosis or had concerns about using technology, and they also added that using the handset may be a burden.

The analysis showed that 62 participants completed the feasibility study. Furthermore, 2 patients were withdrawn during the course of the feasibility study, 1 because of technical difficulties and the other because their chemotherapy treatment was discontinued. Completion of the daily symptom questionnaire on the ASyMS patient handset was high, with patients using it 87.36% (1064/1218) of the time. A 1-way between-groups analysis of variance showed no statistically significant differences in adherence rates (*P*=.15) across countries (United Kingdom=83.1% [349/420], Ireland=90.2% [284/315], Norway=85.7% [60/70], Greece=86.8% [249/287], and Austria=96.8% [122/126]). Similarly, no differences were found in the adherences rates (*P*=.47) for completing the daily questionnaire by cancer type (breast cancer=87.9% [449/511], colorectal cancer=90.7% [400/441], and hematological cancers=80.8% [215/266]).

#### Alert Handling

Across all 13 European cancer centers, a total of 157 amber and 139 red alerts were generated by participants during the feasibility study. Patients with hematological cancers generated an average of 1.25 red alerts per person, those with colorectal cancer had an average of 2.3 red alerts, and those with breast cancer had 2.4 red alerts. Amber alerts followed a similar pattern: patients with hematological cancers generated an average of 2.6 amber alerts, those with colorectal cancer had an average of 2 amber alerts, and those with breast cancer had 2.8 amber alerts.

On an average, it took 38.26 min (SD 138) to handle an amber alert and 15.7 min (SD 20) to handle a red alert. During the monthly trial management meetings, clinicians and researchers across all 5 countries agreed that the timeframe for handling amber alerts (ie, mild to moderate patient symptoms) should be changed from 4 to 8 hours. In addition, clinicians recommended modifications to the ASyMS algorithm regarding the symptom of mucositis (ie, painful inflammation and ulceration of the mouth and throat). It became apparent that clinicians were receiving numerous alerts from patients about mucositis. Even with prompt and appropriate interventions, mucositis takes time to improve. Consequently, patients reported this symptom over multiple days, which triggered an alert to the clinician based on the clinical algorithm. The alert remained active even after it had been handled and patients were given appropriate information and clinical interventions. The algorithm was modified, clinicians were alerted to a patient’s initial report of mucositis, and depending on the severity, subsequent alerts were *silenced* for 1 or 2 days, allowing time for the intervention to relieve symptoms after the alert was initially handled. The modifications required technical changes in the ASyMS and subsequent simultaneous ethical amendments at all participating cancer centers in order to implement the changes.

#### Technical Issues

The ASyMS has a dedicated technical support website for clinicians and researchers to report technical problems and solve issues. This platform allowed users to log, solve, and track issues that arose during the feasibility study. A total of 112 issues were reported during this period. The ASyMS technical support website facilitated rapid and tailored responses, as well as acted as a transparent record of correspondence on technological issues. The most common issues were in relation to using the ASyMS clinician website (31.3% [35/112]), which is the Web-based platform for clinicians and researchers to enroll patients, handle alerts, and monitor feasibility progress. Additionally, 25% (28/112) of the issues were related to the ASyMS clinician handset and 18.8% (21/112) were related to the ASyMS patient handset. All the issues were rectified at each cancer center by the technology partner, who provided additional training on using the system, before progression to the RCT.

### Technological Readiness of the Advanced Symptom Management System at European Cancer Centers

The technological readiness of each cancer center was based on 3 key parameters: system setup, data transfer, and usability issues. Following the completion of the feasibility study, each cancer center was evaluated for readiness to move onto the RCT, using the Technological Feasibility Evaluation Checklists (Multimedia Appendices 1 and 2). Of the 13 cancer centers, 11 passed the technological feasibility evaluation successfully. It was notable that of the 13 cancer centers that completed the feasibility study, 2 reported the intervention was not feasible to integrate into clinical practice (ie, 1 cancer center in the United Kingdom and 1 in Ireland). Both cancer centers were unable to participate because of organizational issues, namely lack of staffing resources to facilitate 24-hour clinician alert handling and technology connectivity issues.

Of the 11 cancer centers that progressed to undertake the RCT, discrepancies existed between reports by the technology company and reports by the cancer centers. Discrepancies included issues involving Wi-Fi and mobile connectivity, local firewall regulations, clinicians’ log-ins, patient enrollment, and completion of patient case note reviews. These issues were investigated and resolved by the researchers at University College Dublin (AB and AD). Following the feasibility study and the evaluation of each cancer center, the principal clinical investigator received a letter from the chief investigator with formal confirmation of permission to progress to the RCT for those 11 cancer centers.

## Discussion

### Principal Findings

This paper details the steps of adapting and implementing a mobile phone–based remote symptom monitoring system at multiple cancer centers across several European countries in preparation for an RCT. Our focus was to outline the complexities involved in preparing, adapting, and implementing an eHealth intervention for an RCT at a multinational scale. The ASyMS has now been adapted and implemented successfully at 11 cancer centers across 5 European countries (ie, Austria, Greece, Ireland, Norway, and the United Kingdom). It is currently being deployed and evaluated in clinical practice at these cancer centers as part of an RCT.

The undertaking of multinational and multicenter eHealth research requires several considerations to address the complexities involved in capturing electronic data [14,34], and researchers in this study faced diverse and unique challenges. While adopting the rigorous and simultaneous steps outlined across Europe, 4 key points of learning emerged, which may provide valuable information for future researchers implementing eHealth studies locally, across cultures and at multiple cancer centers.

Given the multifaceted nature of eHealth [35,36], it was necessary to ensure that the ASyMS was clinically safe and technologically secure at each cancer center before conducting the RCT. Significant time was needed to ensure the European integration of the ASyMS in preparation for its intended RCT. Although the ASyMS was based on preliminary work in the United Kingdom [23-29], the revision and adaptation of the system to make it applicable across multiple European cancer centers involved significant input. Implementation of the ASyMS was achieved through collaborative work with European study partners and a robust, iterative process to resolve problems in each cancer center. Technological Feasibility Evaluation Checklists (Multimedia Appendices 1 and 2) provided effective quality assurance across all cancer centers. The checklists provided a detailed and transparent method of ensuring that each cancer center was suitable to progress to conduct the RCT. The checklists established that the ASyMS was being independently evaluated by clinicians and the technology partner on the same key issues. These enabled the assurance that both clinical and technical issues were being assessed and the issues reported were effectively addressed by the research team. Although 112 issues were reported during the feasibility study, we feel this number is low considering this was across 13 cancer centers and that the technology had not been used before in practice by the clinicians involved. The identification of issues, which could have only been identified through the use of the ASyMS in practice, were not foreseen during the adaption of the system. We would encourage researchers in the field to use and modify the checklists to suit individual study needs, given that each study will have its own set of unique clinical and technical requirements.

As outlined, the questionnaires used in the ASyMS, risk algorithm, and alert management design were refined based on the consultation process, which occurred following the scoping review. This consultation approach aligns with the evidence that advocates the inclusion of clinician and patient consultation is more likely to lead to research that will translate into clinical practice [37,38]. In particular, patient involvement in clinical research is important to ensure that the correct research questions are being asked to address the patients’ and public’s needs [39]. Patient (n=15) and clinician advisory groups (n=21) informed the content of the symptom questionnaires, symptom protocols, clinical algorithms, and self-care advice to ensure consensus across the multiple European cancer centers. The scoping review combined with feedback from clinician and patient advisory groups provided valuable information, which enabled agreement among study partners on the format and content of the intervention, as well as making it current, evidence-based, and culturally sensitive.

Moreover, the content of the ASyMS had to reflect not only current international standards but also be delivered in the appropriate language. A substantial methodological challenge for cross-cultural research is the standardization of the research instruments, particularly the translation of instruments without losing the underlying context or cultural connotations of the wording [40-42]. This process is often time consuming, but it is a crucial investment in order to have confidence in the outcomes of the study. The goal of the translation procedure was to document that each translation adequately captures the concepts of the original English-language version and is readily understood by end users in the target population. We would encourage fellow researchers and developers of eHealth systems, who intend to implement in linguistically varied settings, to factor the time-consuming nature of this step when formulating study timelines and goals. Additionally, when choosing a translation company, we recommend that researchers conduct a scoping exercise of potential candidates to assess their services that will best suit their study’s requirements including a number of criteria: compliance with ISPOR translation and validation guidelines [33]. It is important to consider the company’s experience with translating similar questionnaires and documents with previous research collaborations and completed research projects, reliability and trustworthiness based on testimonials, service costings, and turnaround times to ensure project cost-effectiveness.

The feasibility study of the ASyMS at each cancer center was a crucial methodological step in the transition from its adaptation to implementation into clinical practice. Additional areas were identified where the technology needed to be modified in order to meet the diverse needs of both clinicians and patients. Following the identification of a number of clinical and technical key issues and subsequent discussions at trial management group meetings, the ASyMS was refined and updated to reflect feedback provided by clinicians, researchers, and technological partners. This feedback highlighted that a 4-hour response timeframe was not feasible in busy cancer centers and that the algorithm for the symptom of mucositis warranted modification because of its persistent nature and the amount of alerts clinicians were receiving. Such considerations with the ASyMS algorithm and its related clinician alerts only became apparent during its deployment at multiple cancer centers. Thus, we encourage researchers who intend on conducting multicenter or multinational research using an eHealth intervention to conduct a feasibility study at each intended cancer center, as clinicians and researchers may experience the system differently at each cancer center or country and thus may identify areas of concern. One cannot assume a “one-size-fits-all” model regarding implementing eHealth systems within various clinical settings. In addition, the feasibility study allowed the research consortium to identify cancer centers that were unsuitable to progress to conducting the RCT because of existing heavy workloads and the perceived complexity of the intervention. This echoes the importance of testing an intervention in its intended and various contexts [43], as well as the establishment of communication pathways that clinicians and researchers can use to gain first-hand experience about the system [44].

Successfully implementing new clinical practices in real world settings can be challenging. A significant outcome of the feasibility study was the establishment of relationships and communication between the ASyMS research team and the clinicians at each cancer center. eHealth systems are often predeveloped by researchers to suit a clinical setting and clinicians are asked to assist in effectively implementing them [45]. This approach has been previously criticized as being ineffective in producing effective translation and sustained implementation of evidence-based practices [46]. In the case with the ASyMS, it was vital that strong working relationships and rapports were developed between the research team and the clinicians at the cancer centers. The establishment of relationships between the researchers and clinicians facilitated patient recruitment, since clinicians became aware of the participant criteria and notified the research team when a patient met the inclusion criteria. Additionally, the feasibility study allowed clinicians to become familiar with the study protocol and procedures. For example, when participants were recruited, it was done when they visited the cancer center for chemotherapy treatment where relationships were already developed. On the basis of previous research that showed clinicians’ concern and apprehension about new eHealth technologies [47-49], the feasibility study of the ASyMS helped the research team identify clinicians’ concerns and provide additional training sessions that afforded clinicians the opportunity to learn about the study protocol [30], express their concerns, and ask questions about the technology.

### Limitations

Although these findings may guide future research in multinational eHealth research in cancer care and other areas, the limitations of our approach must also be noted. The cancer centers approached to take part in the ASyMS research were deemed clinically and technologically ready to partake in the research, given that they were teaching hospitals and actively engaged in other research activity. Thus, the implementation and deployment of the ASyMS at these cancer centers may not be representative of other cancer centers that do not have such research and technological resources. The 2 cancer centers that did not proceed to the RCT had intended to implement the ASyMS but did not have the efficient resources (ie, time and staff). Cancer centers that were averse to technology may not be represented in this sample. Also, it must be noted that the feasibility study should be interpreted in the context of another limitation in that patients were not recruited before the initiation of chemotherapy. Therefore, some patients were chemotherapy naïve and others had received previous chemotherapy treatments, which may have affected the results. However, despite these limitations, our work provided significant data around feasibility, changes needed for future use, and the perceived benefits of such a system in cancer centers.

### Conclusions

Patients with cancer who are receiving chemotherapy require prompt identification of symptoms and interventions to decrease the symptom burden and enhance their quality of life. Adapting and implementing a multicenter remote symptom monitoring eHealth system demands significant and substantial collaborative preparatory work across multinational settings before the deployment of an RCT. The findings discussed in this paper outline the importance of effective collaborative project management, diligent use of checklists, clear division of responsibilities with each partner, country, and associated cancer centers, along with addressing cultural and language requisites so that the scientific integrity and reproducibility of the study are assured.

## Appendix

Multimedia Appendix 1 Feasibility evaluation checklist for clinicians.

Multimedia Appendix 2 Technological feasibility evaluation checklist.

## Abbreviations

ASyMS: Advanced Symptom Management System
eHealth: electronic health
eSMART: electronic Symptom Management using Advanced Symptom Management System Remote Technology
ISPOR: International Society for Pharmacoeconomics and Outcomes Research
RCT: randomized controlled trial
