# Supplementary material for: Adaptation and Implementation of a Mobile Phone–Based Remote Symptom Monitoring System for People With Cancer in Europe
Source: JMIR Cancer. 2019 Mar 14;5(1):e10813. doi: 10.2196/10813 (PMC6437605; doi:10.2196/10813)
Supplement: Multimedia Appendix 1 [file cancer_v5i1e10813_app1.pdf]

Multimedia Appendix 1: Feasibility Evaluation Checklist for Clinicians

**Clinical Site**

**Parameters of Effectiveness** Feasibility Parameters (Part 1)
Please complete this form and send it to (insert contact details)

**Have each of the following been completed satisfactorily?**	**Y/N**
Setup	
Training of research nurse / assistant to use ASyMS	
Registration of clinicians on ASyMS	
Patient related	
Registration of patients on ASyMS	
Transfer of data from patient handset to study server (successful connectivity indicated by a green segment in the connectivity history bar)	
Connectivity	
Technological connectivity of ASyMS (mobile connectivity/Wi-Fi/other) - clinician handsets	
Technological connectivity of ASyMS (mobile connectivity/Wi-Fi/other) – tablets	
Technological connectivity of ASyMS (used wireless/wired network connectivity) - system	
Clinician related	
Patients registered on the server have become available on the PROM terminal	
Completion of electronic PROM data by patients and successful transfer to study server	
Completion of electronic Case Note Review data and successful transfer of to study server	

Support system	
Have been able to login to the ASyMS technical support website	

**If "no" was answered to any of the above questions, please provide details:**

**Please sign and date form on page 2**

All clinical sites that meet the afore-mentioned requirements will proceed to Part 2.	
**Docobo name and signature:**	**Date:**
**University College Dublin Researcher name and signature:**	**Date:**
**CI name and signature:**	**Date:**
