# Supplementary material for: Adaptation and Implementation of a Mobile Phone–Based Remote Symptom Monitoring System for People With Cancer in Europe
Source: JMIR Cancer. 2019 Mar 14;5(1):e10813. doi: 10.2196/10813 (PMC6437605; doi:10.2196/10813)
Supplement: Multimedia Appendix 2 [file cancer_v5i1e10813_app2.pdf]

Multimedia Appendix 2: Technological Feasibility Evaluation Checklist

**Clinical Site:**
**PI:**

**Parameters of Effectiveness** Feasibility Parameters (Part 1)
Please complete this form and send it to (insert contact details)

**Have each of the following been completed satisfactorily?**	**Y/N**
Setup	
Training of research nurse / assistant to use ASyMS	
Training of clinicians to use ASyMS	
Registration of clinicians on ASyMS	
Patient related	
Registration of patients on Promasys	
Training of patients to use ASyMS	
Registration of patients on ASyMS	
Completion of electronic clinical and demographic patient data and successful transfer to the study server	
Registration of patients on patient device	
Use of patient device (completion of symptom questionnaire, access to self-care, access to symptom graphs, library, useful contacts, visibility/speech setting) by patients	
Connectivity	
Technological connectivity of ASyMS (mobile connectivity/Wi-Fi/other) - patient devices	
Technological connectivity of ASyMS (mobile connectivity/Wi-Fi/other) – clinician handsets	

Technological connectivity of ASyMS (mobile connectivity/Wi-Fi/other) – tablets	
Clinician related	
Ability of clinicians to log on and use ASyMS clinician handset for the receipt of alerts	
Ability of clinicians to access and log onto the ASyMS web-portal	
Ability of clinicians to deal with an alert using the ASyMS web portal	
Completion of electronic PROMs (pre- and post-CTx assessments) by patients and successful transfer to study server	
Completion of Case Note Review and successful transfer to study server	
Support system	
Ability to access/use the ASyMS technical support website	

If "no" was answered to any of the above questions, please provide details on page 2:

Please sign and date form on page 3

**If "no" was answered to any of the above questions, please provide details:**

All clinical sites that meet the afore-mentioned requirements will proceed to Part 2.	
**PI name and signature:**	**Date:**
**University College Dublin Researcher name and signature:**	**Date:**
**CI name and signature:**	**Date:**
